# Tailoring Subunit Vaccine Immunity with Adjuvant Combinations and Delivery Routes Using the Middle East Respiratory Coronavirus (MERS-CoV) Receptor-Binding Domain as an Antigen

**DOI:** 10.1371/journal.pone.0112602

**Published:** 2014-11-18

**Authors:** Jiaming Lan, Yao Deng, Hong Chen, Guangwen Lu, Wen Wang, Xiaojuan Guo, Zhuozhuang Lu, George F. Gao, Wenjie Tan

**Affiliations:** 1 Key Laboratory of Medical Virology, Ministry of Health, National Institute for Viral Disease Control and Prevention, China CDC, Beijing 102206, China; 2 Department of Pathogenic Biology, Hebei Medical University, Shijiazhuang 050017, China; 3 CAS Key Laboratory of Pathogenic Microbiology and Immunology, Institute of Microbiology, Chinese Academy of Sciences, Beijing 100101, China; Chinese Academy of Medical Sciences, China

## Abstract

The development of an effective vaccine is critical for prevention of a Middle East respiratory syndrome coronavirus (MERS-CoV) pandemic. Some studies have indicated the receptor-binding domain (RBD) protein of MERS-CoV spike (S) is a good candidate antigen for a MERS-CoV subunit vaccine. However, highly purified proteins are typically not inherently immunogenic. We hypothesised that humoral and cell-mediated immunity would be improved with a modification of the vaccination regimen. Therefore, the immunogenicity of a novel MERS-CoV RBD-based subunit vaccine was tested in mice using different adjuvant formulations and delivery routes. Different vaccination regimens were compared in BALB/c mice immunized 3 times intramuscularly (i.m.) with a vaccine containing 10 µg of recombinant MERS-CoV RBD in combination with either aluminium hydroxide (alum) alone, alum and polyriboinosinic acid (poly I:C) or alum and cysteine-phosphate-guanine (CpG) oligodeoxynucleotides (ODN). The immune responses of mice vaccinated with RBD, incomplete Freund’s adjuvant (IFA) and CpG ODN by a subcutaneous (s.c.) route were also investigated. We evaluated the induction of RBD-specific humoral immunity (total IgG and neutralizing antibodies) and cellular immunity (ELISpot assay for IFN-γ spot-forming cells and splenocyte cytokine production). Our findings indicated that the combination of alum and CpG ODN optimized the development of RBD-specific humoral and cellular immunity following subunit vaccination. Interestingly, robust RBD-specific antibody and T-cell responses were induced in mice immunized with the rRBD protein in combination with IFA and CpG ODN, but low level of neutralizing antibodies were elicited. Our data suggest that murine immunity following subunit vaccination can be tailored using adjuvant combinations and delivery routes. The vaccination regimen used in this study is promising and could improve the protection offered by the MERS-CoV subunit vaccine by eliciting effective humoral and cellular immune responses.

## Introduction

In 2012 a novel human coronavirus, Middle East respiratory syndrome coronavirus (MERS-CoV), caused outbreaks of a SARS-like illness in the Middle East, and is now considered a threat to global public health [1], [2]. As of July 23, 2014, the World Health Organization (WHO) reported 837 confirmed cases of MERS-CoV infection, including 291 deaths (a case fatality rate of 34.8%) [3]. Now, studies show that camels are a likely primary source of the MERS-CoV that is infecting humans [4], [5], [6]. But the routes of transmission between camels and people which is the key point to stop transmission of the virus, is far from clearly understood. The continued threat of MERS-CoV necessitates the development of an effective vaccine.

Some studies have indicated that recombinant receptor-binding domain (rRBD) protein of MERS-CoV spike (S) is a good candidate antigen for a MERS-CoV subunit vaccine [7], [8], [9], [10]. However, highly purified proteins are typically not inherently immunogenic, as they usually lack the means to directly stimulate the innate immune system [11]. Besides, they are often prone to degradation. Hence, they call for efficient delivery systems and potent immunostimulants, jointly denoted as adjuvant(s) to evoke the desired antigen-specific immune response phenotype enabling successful vaccination [12].

Aluminium is one of the most common adjuvant in non-living vaccines, has a record of successful use in human vaccination where it promotes antibody-mediated protective immunity [13]. Another classic adjuvant is that based on a water-in-oil-emulsion formulation, such as incomplete Freund’s adjuvant (IFA). Recently, researches have focused on adjuvants that signal through pattern recognition receptors (PRRs), such as Toll-like receptors (TLRs) [14]. Cysteine-phosphate-guanine (CpG) oligodeoxynucleotides (ODNs), which activate B cells and plasmacytoid dendritic cells via TLR9 and induce both innate and adaptive immunity, are currently being developed as a vaccine adjuvant [15]. Another frequently used adjuvant is polyriboinosinic acid (poly(I:C)), a synthetic dsRNA that mimics the effects of naturally occurring dsRNA, a TLR3 agonist [16], [17].

Beside of enhancing the immune response, adjuvant(s) can tailor-make the polarization immune response. For example, ppolarized Th1-type immunity can be achieved by the addition of Freund’s adjuvant or CpG DNA to an antigen. On the other hand, Th2 antibody responses can be induced by the Alum, as indicated by increased IgG1 relative to IgG2a [18], [19]. However, in situations where both Th1 and Th2 responses are required for protection, the choice of one regimen over another might be counter effective. This has led to additional research for alternative adjuvants or adjuvant combinations that promote balanced mixed Th1/Th2 responses [18].

In recent years, the combination of antigens with more than one adjuvant, called the adjuvant system approach has produced vaccines with the ability to generate effective immune responses adapted to both the pathogen and the target population [20]. By using multiple adjuvants in combination, antigen presenting cell (APC) activation is influenced at more than one level, guiding the subsequent adaptive pathways and ultimately inducing a more robust immune response [20].

The induction of a robust humoral, including potent neutralizing antibodies, and cellular immune response is likely essential for immediate and sustained protective immunity in a MERS-CoV vaccine design. In this study, different adjuvants combination regimens including alum, IFA, CpG and poly(I:C) were compared in an effort to promote balance between Th1 and Th2 immune response to bystander rRBD antigen spanning residues 367–606 of MERS-CoV S in a murine model to develop an effective vaccine against MERS-CoV infection.

## Materials and Methods

### Ethics statement

Animal studies were carried out in strict compliance with the Guide for the Care and Use of Laboratory Animals of the People’s Republic of China. The study protocol was approved by the Committee on the Ethics of Animal Experiments of the Chinese Centre for Diseases Control and Prevention. All procedures were performed under ethylether anesthesia and all efforts were made to minimize suffering.

### Vaccine formulation

**Figure 1:**
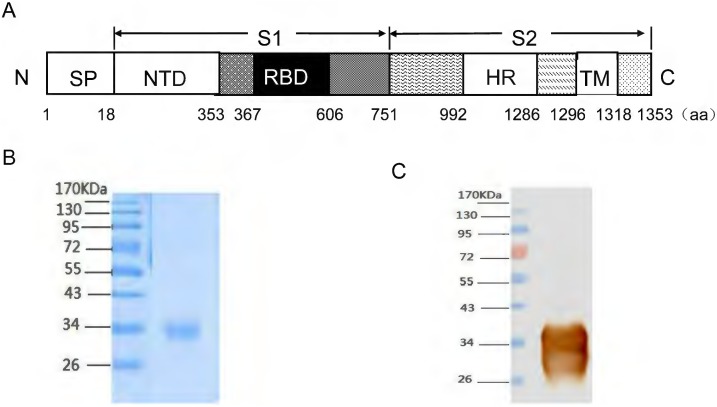
Schematic of the MERS-CoV RBD protein and purification of recombinant RBD protein. A) The location of RBD on the MERS-CoV spike protein. B) SDS-PAGE of purified recombinant RBD protein from a baculovirus expression vector system (BEVS). Lane 1 contains a protein molecular-weight marker, and lane 2 contains purified recombinant RBD protein. C) Western-blot analysis of recombinant RBD protein by a mice polyclonal antibody against spike of MERS-CoV.

MERS-CoV rRBD protein, containing a 240-amino-acid fragment spanning residues 367–606 (Figure 1A) of GenBank number JX869059, was prepared using a Bac-to-Bac baculovirus expression system as described in detail previously [21]. The required rRBD was measured by SDS-PAGE (Figure 1B) and Western Blot (Figure 1C) with a mice polyclonal antibody against spike of MERS-CoV (Figure 1C). Before vaccination, the rRBD protein was quantified by Bradford method.

The rRBD protein was combined with different adjuvants immediately prior to immunisation. Aluminium hydroxide was kindly provided by the North China Pharmaceutical Group Corporation GeneTech Biotechnology Development Company. The ODN motif containing unmethylated CpG (5′-TCCAT-GACGTTCCTGACGTT-3′) was synthesised by TAKARA BIO INC. Poly(I:C) and IFA were purchased from Sigma (St. Louis, MO). A single dose (10 µg) of rRBD protein (100 µL) was combined with either 100 µg of alum alone (RBD/A), alum plus 10 µg of CpG (RBD/A+C), alum plus 50 µg of poly(I:C) (RBD/A+P) or 10 µg of CpG and 100 µl of IFA (RBD/I+C).

### Mouse vaccination and sample collection

**Figure 2:**
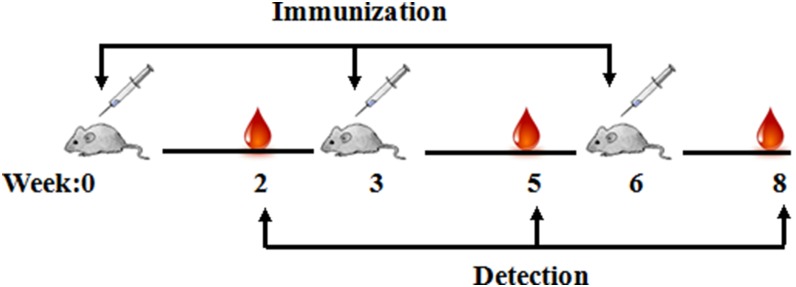
Vaccination and analysis timeline schematic. Eight mice per group received three vaccinations with 10 µg of recombinant RBD protein combined with different adjuvants at 3 weeks intervals. Sera were collected at the indicated times to analyze the humoral immune response. Mice were sacrificed 2 weeks after the last immunisation, and the spleen and lungs were harvested.

Six-to-eight-week-old female BALB/c mice (Animal Care Centre, Chinese Academy of Medical Science, Beijing, China) were randomly distributed into eight groups. Eight mice of each group were vaccinated three times with rRBD proteins at 3-week intervals by either an intramuscular (i.m.) or a subcutaneous (s.c.) route (Table 1). Sera were collected 2 weeks after each vaccination and heat-inactivated at 56°C for 30 min before detection of RBD-specific and neutralizing antibodies. Mice were scarified 2 weeks after the last immunisation, and their lungs and spleens were harvested for detection. A schematic of the vaccination and analysis timeline is shown in Figure 2.

**Table 1:** Vaccination groups and seroconversion after the first and the second immunization.

Groups	Immunogen/dosage	Adjuvant(s)	Route	seroconversion	
				2 weeks	5 weeks
1	–	A*/100 µg	i.m.	0	0
2	rRBD/10 µg	A/100 µg		12.5%	87.5%
3	–	A/100 µg +P*/50 µg		0	0
4	rRBD/10 µg	A/100 µg + P/50 µg		12.5%	100%
5	–	A/100 µg +C*/10 µg		0	0
6	rRBD/10 µg	A/100 µg + C/10 µg		75%	100%
7	–	I*/100 µl + C/10 µg	s.c.	0	0
8	rRBD/10 µg	I/100 µl + C/10 µg		0	100%

A*, Aluminium hydroxide; P*, poly(I:C); C*, CpG; I*, IFA.

### ELISA and IgG isotype ELISA

ELISA was used to detect the MERS-CoV RBD-specific antibody response in immunised mice. Briefly, 96-well ELISA plates were pre-coated with rRBD protein (100 ng/well) overnight at 4°C and blocked with 2% non-fat milk for 2 h at 37°C. Serially diluted sera of eight mice in each group were added to the plates and incubated at 37°C for 1 h, followed by four washes with phosphate-buffered saline (PBS) containing 0.1% Tween 80 (PBST). Bound antibodies were incubated with HRP-conjugated anti-mouse IgG, IgG1, IgG2a or IgG2b (1∶5,000, Sigma) for 1 h at 37°C. The reaction was visualised by using 3, 3′, 5, 5′-tetramethylbenzidine (TMB) peroxidase substrate solution (Invitrogen) and stopped by addition of 2 M H_2_SO_4_. Absorbance at 450 nm was measured using an ELISA plate reader (Wellscan MK 3). The cut-off value was set 2.1-fold above that of the negative control.

### Avidity ELISA

Antibody avidity was determined using the ELISA method described by Vermont et al [22]. Briefly, sera were diluted to a titre of 1∶100, and an ascending concentration of the chaotropic agent NaSCN (0–7 M) was added to the plate. Plates were incubated for 15 min at room temperature (RT) before washing and development to determine total IgG. As a control for antibody specificity, ELISA was used to measure the total anti-MERS-CoV IgG titres of pre- and post-vaccination sera samples.

### Pseudovirus Neutralisation Assay

The conventional neutralization assay using live MERS-CoV is cumbersome and has to be performed in biosafety level-3 facilities. Therefore, we adapted a MERS-CoV pseudovirus system which is sensitive and quantitative, and can be conducted in biosafety level-2 facilities as reported by Zhao et al [23]. In brief, 293T cells were co-transfected with a plasmid encoding codon-optimized MERS-CoV S protein and a plasmid encoding Env-defective, luciferase-expressing, HIV-1 genome (pNL4-3R-E-Luc) using Fugene HD reagents (Roche, Basel, Switzerland). Supernatants containing MERS-CoV pseudovirus were harvested 48 h post-transfection and used for single-cycle infection. Huh7.5 cells were plated at 10^4^ cells/well in 96-well tissue-culture plates and grown overnight. The supernatants containing pseudovirus were pre-incubated with 2-fold serially diluted mouse sera at 37°C for 1 h before addition to cells. The culture was refed with fresh medium 24 h later and incubated for an additional 48 h. Cells were washed with PBS and lysed using lysis reagent included in a luciferase kit (Promega). Aliquots of cell lysates were transferred to 96-well Costar flat-bottom luminometer plates (Corning Costar), followed by addition of luciferase substrate (Promega). Relative light units were determined immediately in the Gaomax luminometer (Promega). All experiments were carried out in triplicate.

Pseudovirus inhibition (PI) rate was calculated as:

(Relative luciferase units of mock sera – relative luciferase units of immune serum for a given dilution)/Relative luciferase units of mock sera.

### ELISpot

To evaluate the antigen-specific T-cell response induced by the vaccination regimes, an IFN-γ ELISpot assay was performed as described previously [17]. Briefly, 96-well plates were coated with 100 µL per well of 5 mg/mL anti-mouse IFN-γ antibody (BD Pharmingen) overnight at 4°C and then blocked for 2 h at RT. Freshly harvested splenocytes (5×10^5^ per well) or lung lymphocytes of eight mice in each group were isolated as described previously [22]. Then, 4 mg/mL of a synthesised 18-mer peptide library, which overlapped the MERS-CoV S RBD by 10 amino acids, was added to the wells in triplicate. Next, a biotinylated detection antibody (BD Pharmingen) and streptavidin-horseradish peroxidase were added. Blots were developed by the addition of an AEC (3-amino-9-ethylcarbazole) substrate solution, which produced a coloured spot after 5-min RT incubation in the dark. Finally, IFN-γ spot-forming cells (SFCs) were counted. Phorbol 12-myristate 13-acetate (PMA) and ionomycin were added to the positive-control group, whereas the negative-control group received no stimuli. The number of peptide-specific IFN-γ secreting T cells was calculated by subtracting the negative-control value from the SFC count.

### Cytometric Bead Array (CBA)

A CBA analysis was conducted to investigate the levels of Th1- and Th2-type cytokine secretion [18] in mice after three times’ immunization. In brief, splenocytes (5×10^5^ per well) of eight mice in each group were distributed in 96-well plates and stimulated with 4****mg/mL of pooled RBD peptide. Plates were incubated for 24****h at 37°C and supernatants were harvested. The concentrations of cytokines, including IL-2, IL-4, IL-6, IL-10, TNF-α, IL-17A and IFN-γ, were measured using a mouse Th1/Th2/Th17 cytokine kit (BD Biosciences) and a FACS Calibur flow cytometer (Becton Dickinson). Data were analysed using the FCAP Array software (Becton Dickinson).

### Statistical Analysis

Statistical analysees were conducted using the one-way ANOVA function in the SPSS 17.0 software package. A *p*-value less than 0.05 were considered to indicate statistical significance.

## Results

### The vaccination regime affects the RBD-specific IgG response in mice

**Figure 3:**
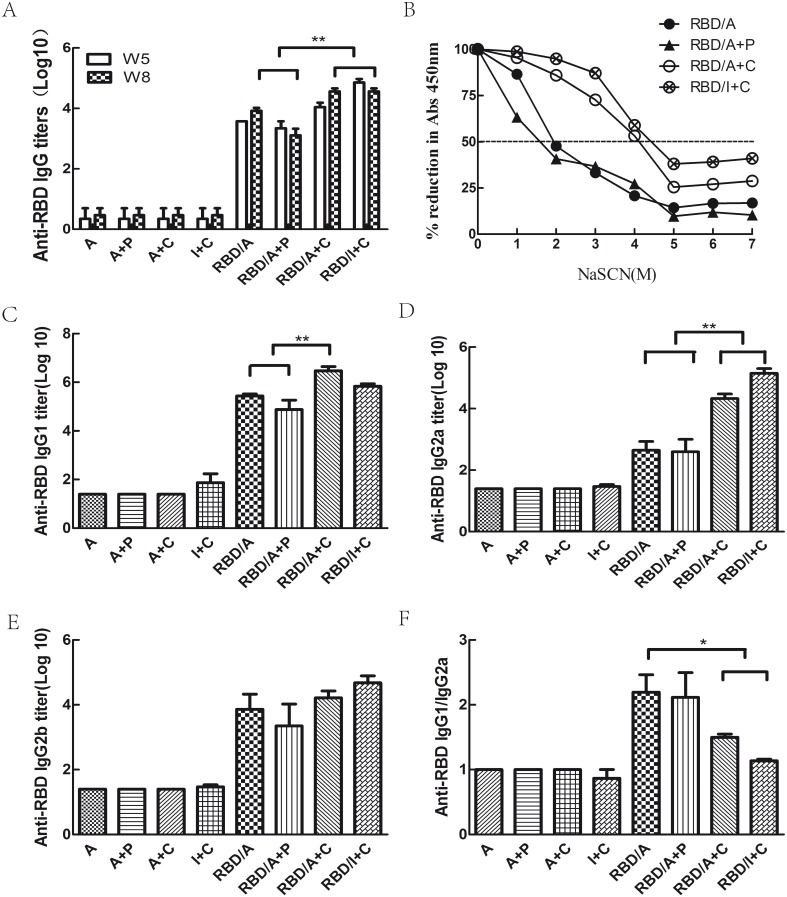
RBD-specific antibody response in immunised mice. A) Total RBD-specific IgG titres were determined by ELISA 2 weeks after the second and third immunisations. B) NaSCN-displacement ELISA to measure the avidity of RBD-specific IgG 2 weeks after the third immunisation. Median displacement is shown for each group against an increasing concentration of NaSCN. The level of NaSCN required to displace 50% of the IgG is indicated. C, D, E and F) Total IgG1, IgG2a and IgG2b RBD-specific titres were determined by ELISA 2 weeks after the third immunisation. Each group contained 8 mice and all experiments repeated for 3 times.

To assess the humoral immune response to different immunisation regimens, mice were immunised with rRBD protein combined with different adjuvants three times at 3-week intervals. Serum samples were collected 2 weeks after each vaccination and total anti-MERS-CoV RBD IgG antibody titres were determined by ELISA. The results indicated that rRBD protein combined with any adjuvant, including alum, IFA, CpG or poly(I:C), could induce a RBD-specific IgG antibody response in the majority of mice after the second immunisation. In a few vaccinated mice, RBD-specific IgG antibodies could be detected even after the first immunisation. The seroconversion rates of the different groups following the first and the second immunisations are shown in Table 1. As shown in Figure 3A, there was no discernible increase in IgG titres after the third immunisation compared to the second immunisation. Among the vaccination regimes, RBD/A+C and RBD/I+C elicited the highest total IgG titres (*p*<0.05, Figure 3A). Besides, the difference of IgG titer in these two groups was not significant (*p*≥0.05, Figure 3A). Similarly shown in Figure 3A, the difference of IgG titer in RBD/A and RBD/A+P groups had no significance. The RBD-specific antibodies were lower than 1∶10 in the adjuvant control groups at each of the three vaccinations.

The responses to the various vaccination regimes were investigated using NaSCN antibody-displacement ELISA to measure antibody avidity (Figure 3B). Mice received the RBD/A+C or RBD/I+C regimes had higher antibody avidity 2 weeks after the final vaccination than those received the RBD/A or RBD/A+P regimes. It was also noteworthy that high antibody avidity correlated with a high IgG titre in mice.

To further characterise the immune response to the different vaccination regimes, IgG isotype analyses were performed 2 weeks after the final vaccination using secondary antibodies against IgG1, IgG2a and IgG2b. As shown in Figures 3C, D, E and F, mice immunised with RBD/A+C or RBD/I+C produced higher IgG1 and IgG2a titres than mice immunised with RBD/A or RBD/A+P. Also, the IgG1 to IgG2a ratio revealed a Th1 skewed response in mice that received the RBD/A+C or RBD/I+C regimes. In contrast, the RBD/A and RBD/A+P regimes produced a higher IgG1/IgG2 ratio, indicating a Th2 response. The titres of RBD-specific IgG2b antibodies, however, were not significantly different among the vaccination groups (*p*≥0.05).

### The adjuvant combination and delivery route affects the neutralizing antibody response to rRBD

**Figure 4:**
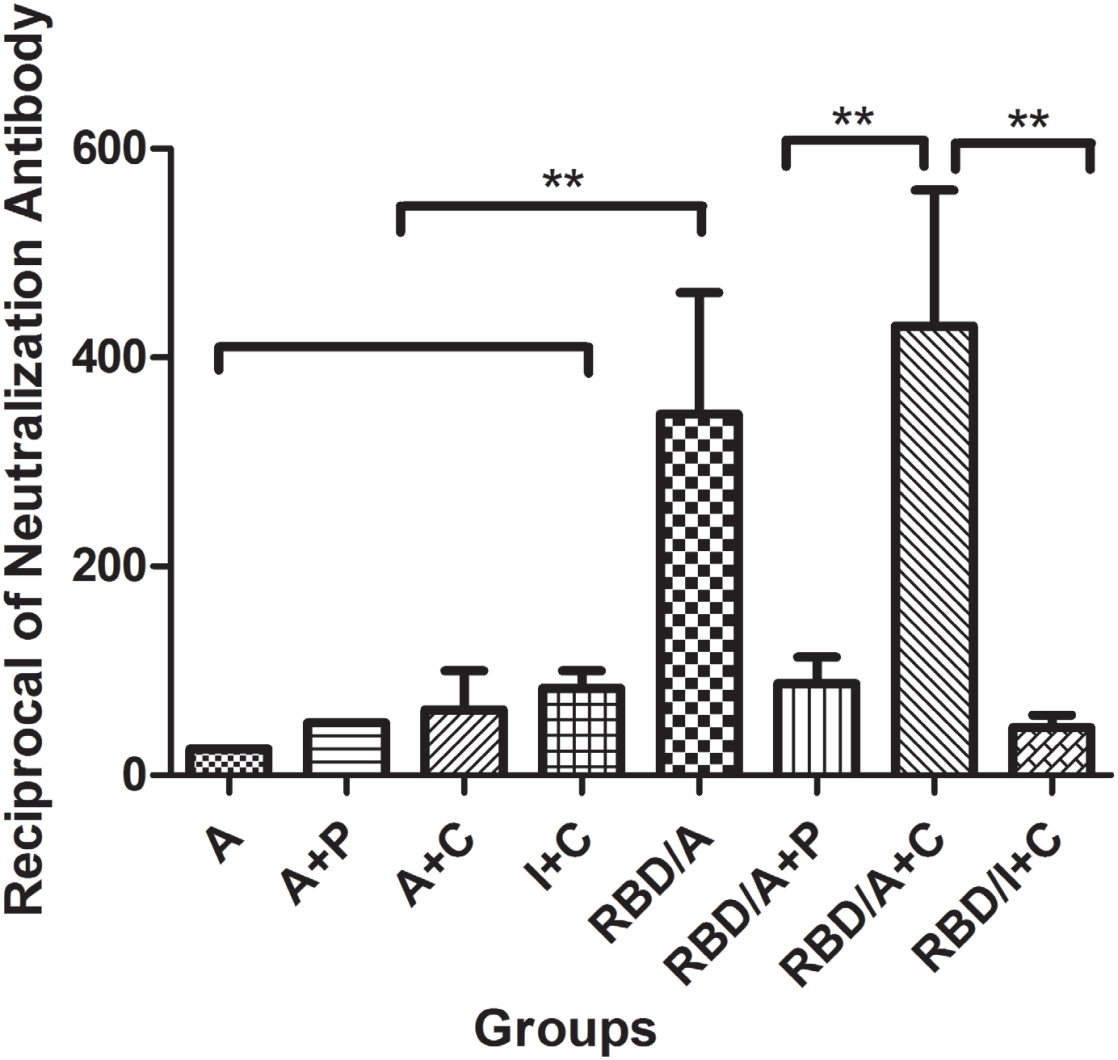
The neutralization antibody responses induced in the immunized mice. Heat-inactived sera after the third vaccination were used for the detection of the neutralization antibodies by MERS-CoV pseudovirus system. Neutralization titres were calculated when the relative inhibition rate was higher than 50%. Data are presented as means with standard error of the mean (SEM). Statistical significance was defined as **p<0.01. A, alum; C, CpG; P, poly(I:C); I, IFA. Each group contained 8 mice and all experiments repeated for 3 times.

Nneutralizing antibodies in the sera of mice immunised with different vaccination regimes were evaluated with a pseudovirus-based neutralization assay. A low level of neutralizing antibodies were detected 2 weeks after the first or second vaccination in aall of the sera tested, although the total IgG antibody levels had almost peaked after the second vaccination. The highest level of neutralizing antibodies was induced after the last vaccination (Figure 4). The pseudovirus inhibition (PI) rates are shown in Figure 4. As shown, the RBD/A+C regime had the highest neutralizing antibody activity (*p*<0.01). Surprisingly, there was low level of detectable neutralizing antibody in the sera of mice immunised s.c. with the RBD/I+C regime, although sera from this group had IgG titres as high as those of the RBD/A+C group after the third immunisation. As expected, no neutralizing antibodies were detected in the sera of mice immunised with the individual adjuvants without rRBD antigen. These results suggest that the MERS-CoV S rRBD protein can induce potent anti-MERS-CoV neutralizing antibody responses when combined with certain adjuvants. Alum and CpG combination maximised the neutralizing antibody response to the rRBD antigen.

### Systematic and local cellular immune response were induced by s.c. vaccination with IFA and CpG combination

**Figure 5:**
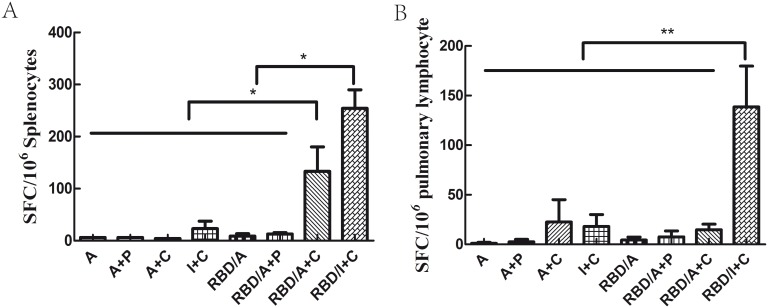
ELISpot analysis of splenocyte and pulmonary lymphocyte IFN-γ secretion. Lymphocytes were isolated 2 weeks after the third immunisation. Data are expressed as spot-forming cells (SFC) responding to RBD-specific peptides and presented as means with a standard error of the mean (SEM). Significant values between the vaccinated groups are defined by *p<0.05 and **p<0.01. Lymphocytes from the spleen (A) and lung (B) of the immunised mice. A, alum; C, CpG; P, poly(I:C); I, IFA. Each group contained 8 mice and all experiments repeated for 3 times.

To characterise the cellular immune responses elicited by the vaccination regimes, single IFN-γ–producing cells were quantified by ELISpot. Both systemic and local cellular immune responses were assessed using lymphocytes from the spleen and lungs of immunised mice. The peptide library used to stimulate the lymphocytes was described in the Materials and Methods section. Results are expressed as the number of SFCs per 10^6^ input cells. Adjuvants without rRBD did not elicit a clear cellular response in the spleen 2 weeks after the third immunisation (Figure 5A). Neither RBD/A nor RBD/A+P induced a significant cellular immune response. In contrast, RBD/A+C and RBD/I+C regimen enhanced a detectable systemic cellular immune response. Furthermore, the RBD/I+C regimen induced the greatest cellular immune response with the greatest number of IFN-γ–producing cells in the spleen (*p*<0.05). A significant cellular immune response in the lung was induced only by the RBD/I+C regime, although a few IFN-γ producing cells were detected in all immunised mice (Figure 5B). We therefore concluded that while both the RBD/A+C and RBD/I+C regimes could induce a systematic cellular immune response in mice, only the RBD/I+C regime could elicit a significant local cellular immune response in the lung.

### A higher frequency of RBD-specific, TNF-α– and IL-4–producing T cells were induced with alum and CpG via an i.m. route

**Figure 6:**
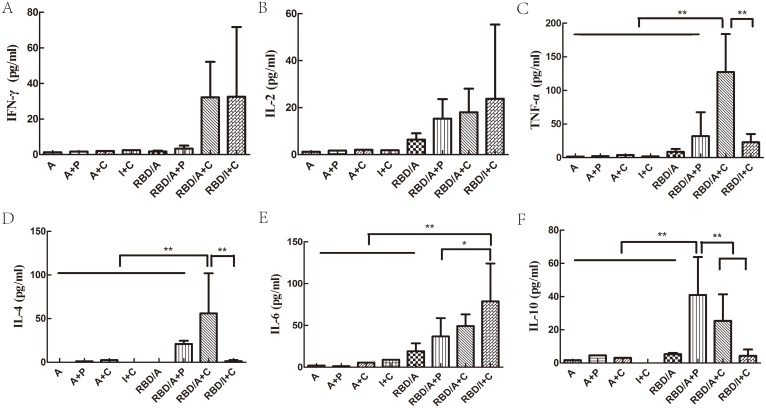
Cytometric Bead Assay (CBA) to determine the in vitro cytokine production of splenocytes from immunised mice after the third immunisation. (A) IFN-γ, (B) IL-2, (C) TNF-α, (D) IL-4, (E) IL-6 and (F) IL-10. Statistical significance was defined by *p<0.05 and **p<0.01. A, alum; C, CpG; P, poly(I:C); I, IFA. Each group contained 8 mice and all experiments repeated for 3 times.

The cytokine profiles of spleen cells from immunised mice were analysed after stimulation with RBD-specific peptides. During CBA, splenocytes from mice immunised with RBD/A+C or RBD/I+C produced IFN-γ (Figure 6A). In contrast, IL-2 was produced by splenocytes following immunisation with rRBD combination of any adjuvants (Figure 6B). But the differences in IFN-γ and IL-2 production among the groups were not significant (*p*≥0.05). Compared with other groups, splenocytes from mice immunised with RBD/A+C induced significantly higher levels of TNF-α (Figure 6C) and IL-4 (Figure 6D) (*p*<0.01**)**. All these indicated that the adjuvants of alum and CpG combination could induce a Th1 and Th2 mixed immune responses in the rRBD antigen model of MERS-CoV, though the responses revealed a Th1 polarization in the isotype ELISA and ELISpot detection. Similarly, the high levels of IFN-γ, IL-2 and IL-6 also indicated a Th1 and Th2 mixed immune responses could be induced by the RBD/I+C regimes. (Figure 6A, 6B, 6E). Different from all of these, as shown in Figure 6F, the RBD/A+P regimes induce the highest level of IL-10 (*p*<0.01), which indicated a Th2 response inclination consistent with the results of IgG isotype. However, IL-17A was not detected in any of the vaccination groups (data not shown).

## Discussion

Coronaviruses can adapt rapidly to new hosts, and an adaptation of MERS-CoV that allowed the virus to efficiently replicate in humans would be a major public health concern, since such an adaptation could trigger a pandemic [24]. The development of an effective vaccine is critical to prevent a potential MERS-CoV pandemic.

Previous studies have shown that vaccination with the SARS-CoV RBD induces highly potent neutralizing antibodies and significantly inhibits SARS-CoV infection [25], [26]. Therefore it was proposed that vaccination with the RBD of MERS-CoV, which belongs to the same *betacoronavirus* genus as SARS-CoV [27], [28], might also inhibit MERS-CoV infection and induce a neutralizing antibody response against MERS-CoV. Du et al [8] identified a recombinant protein containing a 212-amino acid fragment (residues 377–588) in the truncated RBD of MERS-CoV spike protein fused with human IgG Fc fragment (S377-588-Fc) was able to induce in the vaccinated mice strong MERS-CoV S-specific antibodies, which blocks the binding of RBD to dipeptidyl peptidase 4 (DPP4), the human MERS-CoV receptor [29] and effectively neutralizes MERS-CoV infection. Besides, they [9] showed that residues 377 to 662 in the S protein of MERS-CoV induced significant neutralizing antibody responses, suggesting that this region had a potential to be developed as a MERS-CoV vaccine. Mou et al [10] showed the polyclonal antibodies in rabbits against the RBD in the S protein to a 231-amino-acid fragment (residues 358 to 588) efficiently neutralized virus infectivity. However, none of the studies evaluated the immunogenicity of rRBD protein systematically in an animal model. Recently, Ma et al [7] ssuggested the possibility of developing a recombinant RBD protein containing residues 377–662 into an effective and safe mucosal MERS vaccine through the intranasal route in the presence of the only Poly(I:C) adjuvant in a mouse model. While the need for vaccines with the ability to generate an effective immune response has led to the combination of antigens with more than one adjuvant, the ‘Adjuvant System’ approaches. The Adjuvant System approach aids in the development of vaccines that generate effective immune responses [11], [30]. In this study, the roles of three adjuvants–alum, IFA, CpG and poly (I:C)–in rRBD subunit vaccination were investigated aimed at inducing an effective immune response through use of tailored adjuvant combinations and delivery routes.

Consistent with above studies, all vaccination regimes containing rRBD induced an RBD-specific cellular and humoral immune response. However, a more robust immune response was elicited when mice were immunised with the RBD/A+C and RBD/I+C regimes. An unexpected result was the absence of neutralizing antibodies in the sera of RBD/I+C immunised mice, despite anti-RBD specific IgG titres being similar for the RBD/I+C and RBD/A+C regimes. To further understand the riddle, we detected the aantibody avidity of different vaccination regimes by aavidity ELISA. However, the results showed the high antibody avidity correlated with a high IgG titre in mice of RBD/I+C and RBD/A+C groups. So, we speculated maybe the adjuvants of destroyed the conformation of rRBD and covered the antigen binding sites. Another probable cause of the low titer of neutralizing antibodies in the sera of RBD/I+C immunised mice was the delivery route of subcutaneous. As known, the subcutaneous may be associated with degradation at injection site, which leads to decreased bioavailability [31]. Whatever, further studies are in process.

Compared with other studies, the regimes in this study induced lower titres of neutralization antibodies. For example, the PI_50_ of alum plus CpG, the group showing the highest titer of neutralizing antibody in all immunization groups was 1∶500. While the rRBD protein in the above studies acquired a 1∶1000 in mice neutralization antibody titre. The differences may be caused by the detection methods of neutralization antibody. As showed in the materials and methods parts, the neutralization antibodies in this study were detected by a pseudovirus system which can be conducted in biosafety level-2 facilities. While the differences of induced neutralization antibodies among different groups can be shown clearly.

The subclass of immunoglobulin induced after immunization is an indirect measure of the relative contribution of Th1-type cytokines vs. Th2-type cytokines [18]. To characterise the immune response of the different vaccination regimes, IgG isotype including IgG1, IgG2a and IgG2b analyses were performed. As expected, the RBD/A regimes produced a Th2 response with high IgG1/IgG2 ratio. In contrast, mice received the RBD/A+C or RBD/I+C regimes revealed a Th1 skewed response. Consistently, the RBD/A+C or RBD/I+C regimes induced a systematic cellular immune response in mice by ELISpot analysis. The high level of IFN-γ and IL-2 in the CBA was also a proof of the cellular immune response in mice. Besides, the mice in the RBD/A+C group had a high level of IL-4 and IL-10, which were an index of Th2 skewed response. Taken together, the RBD/A+C induced a Th1 and Th2 mixed immune responses, though the responses had a Th1 inclination. It was our original intention of mixed Th1/Th2 responses for better protection. Similarly, the RBD/I+C regimes induced a mixed Th1 and Th2 responses. However, it was a pity that the RBD/I+C regimes could not induce an effective neutralization antibody, which was the most important factor of a prophylactic vaccine.

Above all, in this study, MERS-CoV S rRBD combined with the adjuvants alum and CpG produced the most robust immune response. It indicates that the combination of alum and CpG was the optimal strategy for i.m. rRBD antigen delivery in a murine model. This result will facilitate future MERS-CoV vaccine design. The results of the present study also support the importance of the Adjuvant System approach, although adjuvant combinations do not always produce the desired response, as seen with RBD/I+C. Consistent with the results of the present study, CpG plus alum was found to induce protective humoral, as well as cellular immunity, in mice immunised with a recombinant haemagglutinin vaccine that protected against influenza virus challenge [32]. The ideal immunity of the CpG and alum combination may be the result of mutual complementation of these two adjuvants. It is well known that alum can promote antibody-mediated protective immunity. However, alum is a poor inducer of cellular immune responses [12]. Recently, adjuvants including oil-in-water emulsions have shown improved efficacy for avian influenza protection suggesting that even for diseases where humoral immunity can confer protection, cellular immune responses may be necessary in vaccine design [33]. The key features of CpG-ODN used as a vaccine adjuvant, include the ability to elicit Th1 cell, but only under certain conditions, CD8+ cytotoxic T cell responses and an additional ability to divert the pre-existing Th2 response in neonates and elderly mice toward a Th1 phenotype [34].

Thus, we expect that the combination of alum and CpG will prove applicable in a range of infectious diseases that have defeated current immunisation strategies. Except for a choice of adjuvants in combination with optimal protective antigen, practical items such as the antigen: adjuvant ratio, dose, vaccination regimen and often route of administration will strongly impact on both the effectiveness and safety of the vaccine formulation. In most cases, an experimental vaccine will be initially tested in an animal model [29]. To evaluate the immunogenicity of rRBD protein thoroughly, it is necessary to test the protective effects of rRBD subunit immunisation in an animal model of MERS-CoV infection. To date, rhesus macaques have been reported to generate pneumonia-like symptoms within 24 h of MERS-CoV infection [35], and we are testing the effects of rRBD immunisation in rhesus macaques. Considerable efforts are being made to establish a small animal model of MERS-CoV infection. Though the lung cells of the Syrian hamster express the receptor for MERS-CoV, they are not susceptible to MERS-CoV infection [35], [36]. Recently, a mouse model of MERS-CoV infection was reportedly generated by transduction of mice with adenoviral vectors expressing DPP4 [24]. In the future, we expect the protective effect of the RBD/A+C vaccination should be investigated in this murine model of MERS-CoV infection.
